# CD300c is uniquely expressed on CD56^bright^ Natural Killer Cells and differs from CD300a upon ligand recognition

**DOI:** 10.1038/srep23942

**Published:** 2016-04-04

**Authors:** Milena Dimitrova, Olatz Zenarruzabeitia, Francisco Borrego, Venkateswara R. Simhadri

**Affiliations:** 1 Division of Biotechnology Review and Research-I, Office of Biotechnology Products Review and Research, CDER, Food and Drug Administration, USA; 2 Immunopathology Group, BioCruces Health Research Institute, Barakaldo, Spain; 3 Immunotherapy Group, Basque Center for Transfusion and Human Tissues, Galdakao, Spain; 4 Ikerbasque, Basque Foundation for Science, Bilbao, Spain

## Abstract

Paired receptors on NK cells recognize similar ligands with varied strength of binding ability and perform different functions. The CD300 molecules are emerging as novel immune regulators in health and disease due to their interaction with their lipid-nature ligands. Particularly, the paired receptors CD300c and CD300a have been shown to elicit activating and inhibitory capabilities, respectively. In the current study, we seek to investigate the expression and function of CD300c on human NK cells. We demonstrate that IL-2 and IL-15 treatment significantly induce CD300c expression exclusively on CD56^bright^ NK cells. CD300c up-regulation requires STAT5 and its expression is inhibited by IL-4. Consistently, IL-2 secreted from activated CD4^+^ T cells specifically induces the expression of CD300c on CD56^bright^ NK cells. Crosslinking CD300c with a specific antibody enhances the proficiency of CD56^bright^ NK cells to degranulate and induce chemokine and cytokine secretion. We also show the differential binding of CD300a and CD300c to their ligands phosphatidylethanolamine (PE) and phosphatidylserine (PS) and their differential ability to affect CD56^bright^ NK cell functions. Our results provide an insight into the novel set of paired receptors CD300a and CD300c that are distinctively expressed on CD56^bright^ NK cells with varied effector functions.

Natural Killer (NK) cells are known for their pivotal role in the innate immune system; displaying natural cytotoxicity against tumor-transformed and virus-infected cells, as well as secreting immune-regulatory cytokines^1^,^2^,^3^. Their function is regulated by a multitude of both activating and inhibitory receptors^4^,^5^. Complex interactions of different cellular targets with ligands for both types of receptors determine NK cell inhibition (tolerance) or activation (missing self and stress-induced self). In addition, cytokines such as IL-12, IL-15, IL-18 and IL-1β secreted from monocytes, macrophages and dendritic cells (DC) are primary signals that activate NK cells^6^,^7^,^8^,^9^. In recent years, the importance of NK cell-mediated regulation of adaptive immune responses has also been explored in various scenarios, such as in NK-DC cross talk, the interaction with antigen presenting cells and also through the effect that they have in modulating T and B cell responses^7^,^10^,^11^,^12^,^13^,^14^. Moreover, it has been shown that stimulatory signals like IL-2 from the adaptive immune system (antigen-specific T cells) activate the CD56^bright^ NK cell subset in secondary lymphoid organs and is able to modulate its effector functions^15^,^16^.

Human NK cells are phenotypically characterized by the expression of CD56 and lack of CD3 on their cell surface. Examining the surface density of CD56 expression, NK cells are divided into two distinct subsets, CD56^bright^ and CD56^dim^. In the periphery, approximately 90% of human NK cells are CD56^dim^ expressing high levels of CD16 (FcγRIII) and are predominantly cytotoxic in function. In contrast, only 5–10% of NK cells are CD56^bright^ and CD16^dim/neg^ with a predilection for secreting pro-inflammatory cytokines^17^,^18^,^19^,^20^. Similar to their varied differences in functions, these two subsets express a different array of receptors on their surface, which include activating and inhibitory receptors, adhesion molecules and chemokine receptors^21^,^22^,^23^. Some of these variations determine the homing of NK cells to different lymphoid tissues. For example, CD56^bright^ NK cells home to the secondary lymphoid organs, where they comprise roughly 90% of the NK cell population^15^. Furthermore, CD56^bright^ and CD56^dim^ cells differ in their response to IL-2 for proliferation. CD56^bright^ cells constitutively express high levels of both high and intermediate-affinity IL-2 receptors on their surface, which allow them to proliferate even under low concentrations of IL-2^24^,^25^,^26^. Similar to IL-2, IL-15 also binds with high affinity to the hetero-trimeric receptor complexes, which consist of IL-2/15Rβ (CD122), the common chain (γc or CD132), and IL-15Rα^9^,^15^,^27^. The γc is the main component that transduces the signal via Janus tyrosine-kinase (JAK)-3 to phosphorylate further downstream signaling molecules like signal transducer and activator of transcription (STAT) molecules. This signaling is specific to each receptor complex. In this case, IL-2 and IL-15 mainly activate STAT5 to induce cellular functions such as activation, proliferation and also regulate the receptor repertoire of NK cells^27^,^28^.

The human CD300 family of receptors is a group of eight type-I membrane glycoproteins that harbor a single IgV-like extracellular domain and regulate a diverse array of immune processes. This family is clustered on chromosome 17. Seven members (CD300 a-h) are expressed on leukocytes^29^,^30^. The eighth member, CD300g, is found only on endothelial cells^31^. The human activating receptors, CD300b, CD300c, CD300d, CD300e and CD300h associate with different adaptor molecules such as FcεRIγ chain, DNAX-activating protein (DAP)-12 or DAP10 through their charged residues in the trans-membrane domain. In contrast, the human inhibitory receptors, CD300a and CD300f, elicit inhibitory signals via their immuno-receptor tyrosine-based inhibitory motifs (ITIMs) in the cytoplasmic tail^29^. The ligands for this family of receptor are mostly of lipid nature, including phosphatidylserine (PS) and phosphatidylethanolamine (PE), two amino-phospholipids that are expressed on the outer leaflet of the plasma membrane of dead and activated cells^32^,^33^. The highly homologous members CD300a and CD300c are considered as paired receptors with inhibitory and activating roles, respectively^34^. Although transcripts encoding these receptors were reported in cells from both myeloid and lymphoid lineages, the receptor expression of CD300a and CD300c are indistinguishable on the cell surface due to the lack of specific antibodies. However, recently we have shown that a specific antibody clone, TX45, recognizes CD300c but not CD300a^35^.

Previously, the role of CD300a on NK cells has been investigated to elicit signals to inhibit target cell killing by NK cells^36^,^37^. However, the expression and function of CD300c on NK cells has yet to be elucidated. Here, we show that expression of CD300c is induced uniquely on CD56^bright^ NK cells upon treatment with either IL-2 or IL-15. In addition, we demonstrate that its expression is inhibited in the presence of IL-4 and regulated by the transcription factor STAT5. We also show that cross-linking of CD300c with a specific monoclonal antibody enhances effector functions (degranulation and cytokine secretion) of CD56^bright^ NK cells. Finally, we demonstrate the differential binding of CD300a and CD300c to their ligands PE and PS, and reveal the distinct response of CD56^bright^ NK cells after interacting with these ligands. Our results provide an insight into a novel set of paired receptors - CD300a and CD300c - that are expressed on CD56^bright^ NK cells.

## Results

### CD300c is exclusively expressed on CD56^bright^ NK cells

**Figure 1:**
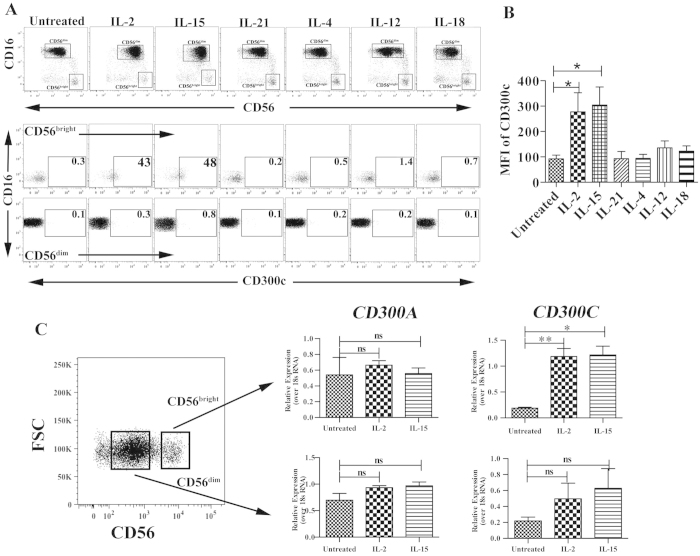
CD300c is exclusively expressed on CD56^bright^ NK cells. (**A**) The gating strategy of NK cells is shown to distinguish the CD56^bright^ and CD56^dim^ populations (upper panel). The dot plots in the bottom panel indicate the percentage of CD300c+ NK cells upon various cytokine stimuli for 40 hours. (**B**) The bar graph shows significant up-regulation of CD300c expression on CD56^bright^ subsets that are stimulated with IL-2 and IL-15. The data are from independent experiments from 4–6 donors. (**C**) The specific mRNA transcripts of *CD300A* and *CD300C* were determined by RT-PCR analysis on the sorted populations of different subsets of unstimulated, IL-2 and IL-15 stimulated NK cells. The y-axis denotes the normalized expression over 18sRNA and the corresponding ΔΔCt values are plotted. The data is from four healthy donors. The error bars indicate the average ± SEM.

Recently, we demonstrated that CD300c is expressed on human monocytes using the specific monoclonal antibody clone TX45^35^. To assess the expression of CD300c on NK cells, we used the same antibody and investigated whether cytokine stimulation influences the expression of CD300c. We stimulated with a wide variety of cytokines that activate NK cells and confirmed that IL-2 and IL-15 significantly up-regulate CD300c expression solely on the CD56^bright^ (CD56^high^ CD16^neg^) subset. In contrast, no expression was detected on CD56^dim^ (CD56^low^ CD16^pos^) subset of NK cells. When NK cells were treated with IL-4, IL-12, IL-18 and IL-21, we observed that CD300c expression was not changed on either of the NK cell subsets (Fig. 1A,B). Moreover, within the CD56^bright^ NK cell subset a small population of CD56^high^ CD16^dim^ NK cells also expressed CD300c upon IL-2 and IL-15 treatment (Supplementary Figure). Since CD300c expression is dependent on IL-2, we tested its expression on NK cells after a prolonged treatment of IL-2 for 7 days mimicking a phenomenon of lymphokine-activated killer (LAK) cells. We show that CD300c expression is maintained only on CD56^bright^ subsets but not on CD56^dim^ subset (Supplementary Figure). To further confirm that the expression of CD300c is transcriptionally regulated, we sorted the two NK cell subsets and analyzed the respective mRNA levels after treatment with IL-2 and IL-15. Previously, we and others have demonstrated that CD300a is expressed on the cell surface of all NK cell subsets^35^,^36^, therefore we chose CD300a as a control. We confirmed that transcripts encoding CD300c were significantly up-regulated in the CD56^bright^ NK cells upon cytokine stimulation but not on CD56^dim^ NK cells. On the contrary, IL-2 and IL-15 stimulation did not have any impact in the transcript levels encoding CD300a (Fig. 1C). These results indicate that expression of the paired receptors CD300a and CD300c are differentially regulated on CD56^bright^ NK cells.

### Expression of CD300c is regulated by STAT5 and IL-4

**Figure 2:**
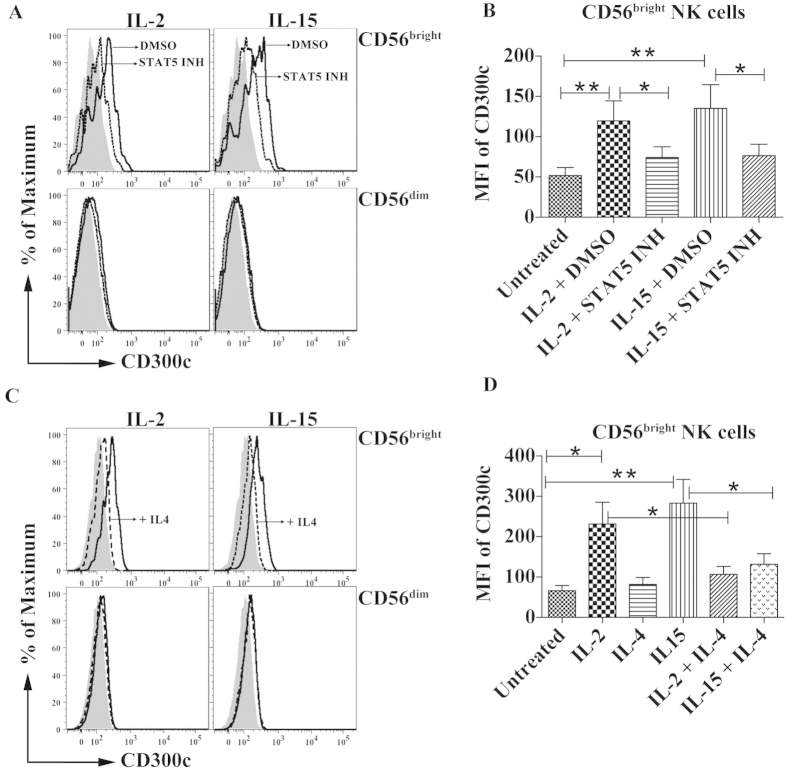
Expression of CD300c is regulated by STAT5 and IL-4. (**A**) Purified NK cells were stimulated with either IL-2 or IL-15 in the presence or absence of STAT5 inhibitor. Representative histograms show the expression of CD300c on different NK cell subsets that are either untreated (shaded), IL-2 or IL-15 plus vehicle DMSO as represented (thick line) and IL-2 or IL-15 plus STAT5 inhibitor (dotted line). (**B**) Bar graph represents the MFI of CD300c expression on CD56^bright^ NK cells with indicated treatments. (**C**) Representative histograms show the expression of CD300c on different NK cell subsets that are either untreated (shaded), IL-2 or IL-15 as represented (thick line) and IL-2 or IL-15 with IL-4 (dotted line). (**D**) Bar graph represents the MFI of CD300c expression on CD56^bright^ NK cells with indicated treatments. The data are from independent experiments from 4 donors. The error bars indicate the average ± SEM.

One of the major mechanisms that are triggered by IL-2 and IL-15 is the activation of STAT5^27^. To determine the role of the latter, we treated NK cells with an inhibitor, N′-((4-Oxo-4H-chromen-3-yl)methylene)nicotinohydrazide, that blocks the biological function of STAT5 in the presence of either IL-2 or IL-15, and we observed that the expression of CD300c was significantly reduced on the CD56^bright^ NK cell subset (Fig. 2A,B). In T cells, it was demonstrated that IL-4 inhibited IL-2-triggered STAT5 activation through a mechanism that involves down-regulation of the kinase activity of JAK1 and JAK3^38^. Therefore, we studied the role of IL-4 in regulating IL-2 and IL-15 mediated expression of CD300c. Inclusion of IL-4 into the culture in conjunction with either IL-2 or IL-15 significantly decreased CD300c expression on CD56^bright^ NK cells when compared with IL-2 or IL-15 treated cells (Fig. 2C,D). Therefore, our results indicate that CD300c expression on CD56^bright^ NK cells is dependent on STAT5 activation and negatively regulated by IL-4.

### T cell-derived IL-2 induces the expression of CD300c on CD56^bright^ NK cells

**Figure 3:**
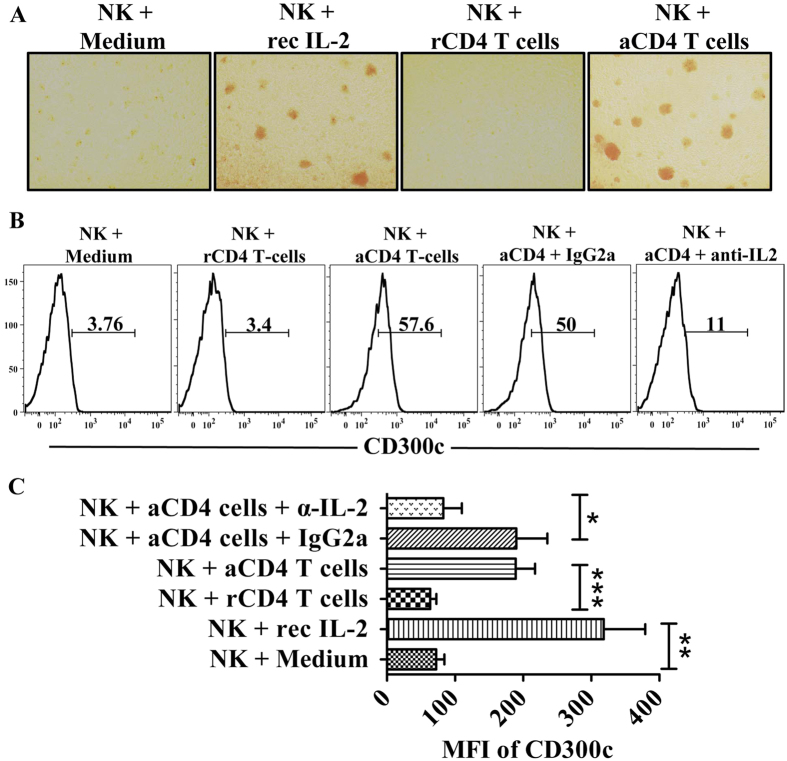
T cell-derived IL-2 induces the expression of CD300c on CD56^bright^ NK cells. (**A**) NK cells form clusters when cultured either in the presence of recombinant IL-2 (rIL-2) or activated CD4^+^ T cells in a trans-well assay. (**B**) The histograms represent the percentage of CD300c^+^ CD56^bright^ NK cells cultured under several conditions in the transwell assay as described in Materials and Methods. (**C**) Bar graph represents the MFI of CD300c expression on CD56^bright^ NK cells cultured under the indicated conditions in the transwell assay. The data are from independent experiments from 4 donors. The error bars indicate the average ± SEM. rCD4 T-cells: resting CD4+ T cells; aCD4 T-cells: activated CD4+ T cells; α-IL-2: neutralizing anti-IL-2 mAb; rec IL-2: recombinant IL-2.

Many studies have shown that CD56^bright^ NK cells localize in the secondary lymphoid organs and play an important role in the ongoing adaptive immune responses^9^. During these responses, APC-activated T cells secrete IL-2 and eventually interact with the high affinity IL-2 receptor present of CD56^bright^ cells enhancing the NK and T cell cross talk^15^. We thus examined the expression of CD300c on CD56^bright^ NK cells in a transwell assay with CD4^+^ T cells as their counterpart. In our experiments, we purified autologous naïve CD4^+^ T cells and NK cells from the same donor. Expanded and activated CD4^+^ T cells were obtained using anti-CD3/CD28 beads. Similar to the results observed with recombinant IL-2, the morphology of the NK cells is more clustered when cultured in the presence of activated CD4^+^ T cells suggesting that NK cells are more activated. This phenomenon is not observed when NK cells were incubated with either medium alone or resting CD4^+^ T cells (Fig. 3A). Then, we determined the expression of CD300c on CD56^bright^ NK cells when cultured with resting and activated CD4^+^ T cells. As expected, CD300c expression is enhanced and this effect was inhibited only when a specific antibody was used to block the biological activity of IL-2. The isotype control did not show any effect. On the other hand, medium alone and resting CD4^+^ T cells did not induce any change in CD300c expression (Fig. 3B,C). Thus, the IL-2 secreted from activated CD4^+^ T cells specifically upregulates CD300c expression on CD56^bright^ NK cells.

### Crosslinking of CD300c induces CD56^bright^ NK cells to degranulate and secrete cytokines

**Figure 4:**
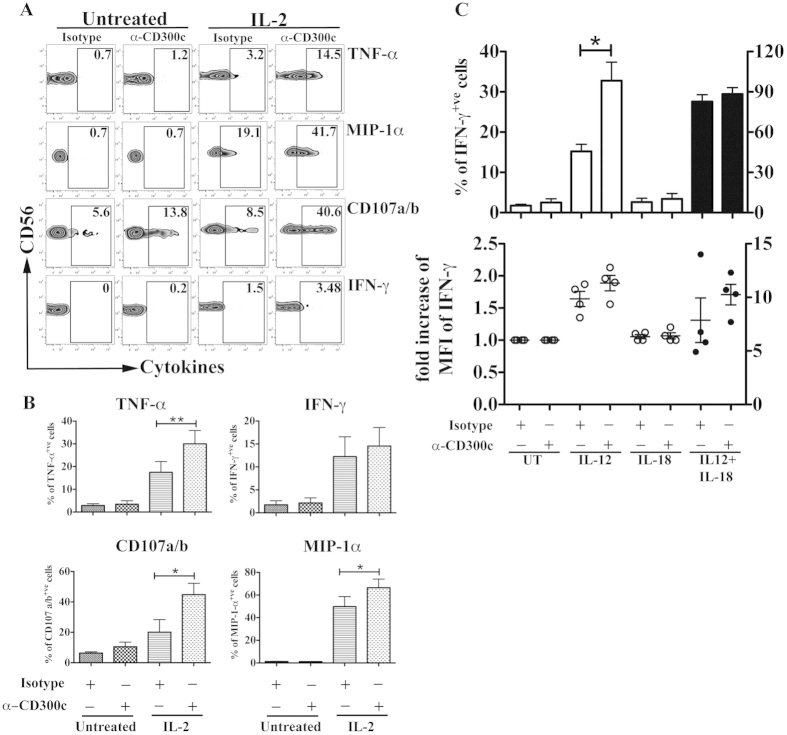
Crosslinking of CD300c induces CD56^bright^ NK cells to degranulate and cytokine secretion. Purified NK cells were both left untreated or pre-treated with IL-2 and cross-linked with isotype or anti-CD300c (clone TX45) to assess NK cell functions. (**A**) The zebra plots represent the production of various cytokines and degranulation markers (CD107a/b) from CD56^bright^ NK cells, gated as in Fig. 1 (gated as CD56^bright^ CD16^neg^). (**B**) Bar graphs represent the percentage of CD56^bright^ NK cells producing cytokines and expressing CD107a/b after different stimulation conditions. The data are from independent experiments from at least 4 donors. (**C**) Purified NK cells were both left untreated or pre-treated with IL-2 and crosslinked with isotype or anti-CD300c (clone TX45), and in the absence or presence of IL-12 and IL-18. The upper panel shows the percentage of IFN-γ^+^ CD56^bright^ NK cells. The lower panel represents the fold increase in the MFI IFN-γ^+^ CD56^bright^ NK cells. The Y axis on the left correspond to the white bars and the white symbols, while the Y axis on the right is for the black bars and the black symbols. The data are from independent experiments from 4 donors. The error bars indicate the average ± SEM.

To determine whether the expression of CD300c on CD56^bright^ NK cells possesses a functional role, CD300c was crosslinked with the specific monoclonal antibody clone TX45^35^, and assessed for the ability of NK cells to degranulate and produce cytokines. Crosslinking of IL-2 pre-activated NK cells with TX45 significantly increased NK cell degranulation, as measured by the expression of CD107a/b and induction of the pro-inflammatory cytokine TNF-α and chemokine MIP-1α (Fig. 4A,B), when compared with only IL-2 pre-activated NK cells. On the other hand, cross-linking of CD300c in IL-2 non-treated NK cells did not show any significant effects.

Although we observed a small percentage of IFN-γ producing cells after IL-2 pre-activation, the effect of CD300c mediated signaling of IFN-γ secretion in IL-2 activated CD56^bright^ NK cells was not clear and pronounced. Because IL-12 and IL-18 are important monokines for IFN-γ production by NK cells^39^, we hypothesized that crosslinking of CD300c may synergize with IL-12 and/or IL-18 in inducing IFN-γ production by IL-2 pre-activated CD56^bright^ NK cells. Therefore, in a different set of experiments, purified NK cells were treated with IL-2 to up-regulate the expression of CD300c and then crosslinked with anti-CD300c monoclonal antibodies in the presence or absence of IL-12 and IL-18. While IL-12 alone can induce IFN-γ secretion, we found a significant increase when NK cells were simultaneously crosslinked with anti-CD300c antibody (Fig. 4C-top). These effects were not observed when cells were crosslinked with anti-CD300c monoclonal antibodies in the presence of IL-18. When NK cells were stimulated with both monokines, there was no significant effect of CD300c mediated-signals on the number of IFN-γ secreting cells, but we observed an enhanced secretion of IFN-γ per cell basis as shown by an increase in the median fluorescence intensity (MFI) of IFN-γ producing cells (Fig. 4C-bottom). Thus, the activating receptor CD300c has a co-stimulatory role for IFN-γ production by CD56^bright^ NK cells.

### Differential binding of paired receptors CD300a and CD300c to PE and PS

**Figure 5:**
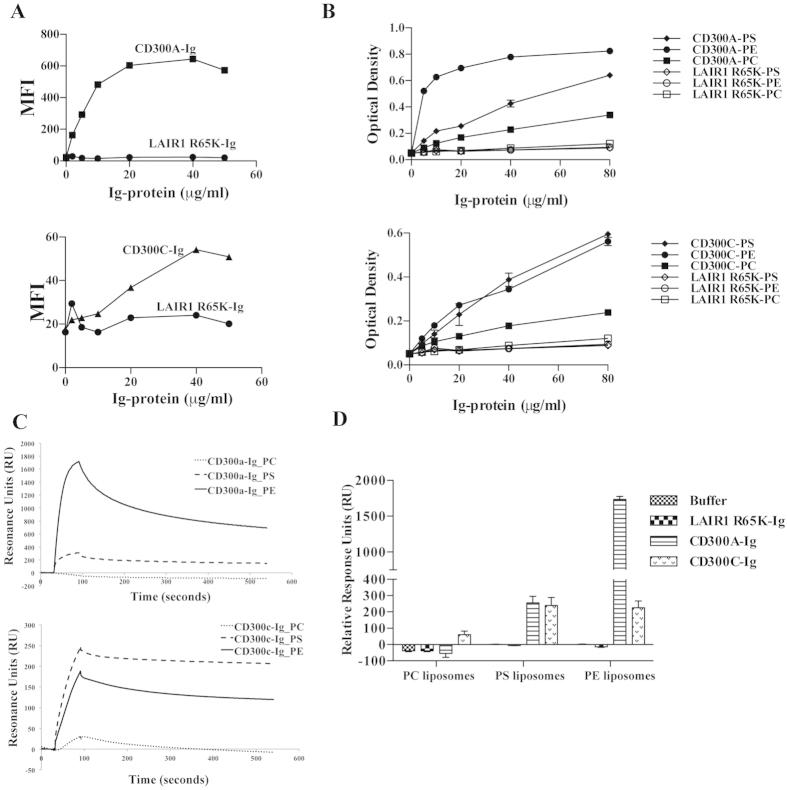
(**A**) Binding of the purified fusion proteins CD300a-Ig (top graph) and CD300c-Ig (bottom graph) to dead Jurkat cells. Increasing concentrations of fluorochrome labeled CD300a-Ig and CD300c-Ig proteins were incubated with dead Jurkat cells and then acquired in a flow cytometer. LAIR1 R65K-Ig fusion protein served as negative control. Graphs represent the binding (MFI) of the fusion proteins to dead cells. The data are representative of two independent experiments. (**B**) ELISA assay shows binding of increasing concentrations of CD300a-Ig (top graph) and CD300c-Ig (bottom graph) fusion proteins to pure lipids that are coated on plates. LAIR1 R65K-Ig fusion protein served as negative control. The data is a representative of 2 independent experiments. (**C**) Binding of CD300-Ig fusion proteins to liposomes. Liposomes of specified compositions were prepared and coupled to a L1 biosensor. The binding of CD300a-Ig (top) and CD300c-Ig (bottom) was analyzed by allowing the proteins to pass through the L1 sensor. The curves represent the binding of fusion proteins to liposome coated L1 chips. (**D**) The binding (RU) for plateau values is shown in the bar graph and the error bars represent the average ± SEM. LAIR1 R65K-Ig fusion protein served as negative control. Results shown are from 3 independent experiments.

In our previous publication we have demonstrated that CD300a interacts with PE and PS exposed on the outer leaflet of the plasma membrane of dead cells^32^. Since the extracellular domain of CD300c is highly homologous to CD300a we hypothesized that CD300c might have a similar binding pattern to these two amino-phospholipids. Therefore, in this report we examined the ability of CD300c to bind PS and PE using multiple assays. For this analysis, we generated a chimeric protein consisting of CD300c extracellular domain and the Fc fragment of human IgG2. Consistent with our reported results^32^, we observed that the fusion protein CD300a-Ig binds to dead cells in increasing concentrations and the binding is saturated at 20 μg/ml; while the negative control did not show any binding (Fig. 5A-top). Similarly, CD300c-Ig also binds to dead cells in a concentration-dependent manner but to a lesser extent compared to CD300a-Ig (Fig. 5A-bottom). Since the plasma membrane asymmetry on the dead cell surface exposes predominantly the amino-phospholipids PS and PE^40^, we next assessed the ability of these two receptors to bind each amino-phospholipid separately. We tested the binding of fusion proteins to pure lipids in a concentration dependent manner. While CD300c-Ig binds to both PS and PE at the same level, CD300a-Ig predominantly binds to PE (Fig. 5B). The negative control protein LAIR R65K-Ig did not show any binding to any of the lipids. The relevant binding of fusion proteins to the negative control lipid phosphatidylcholine (PC) is minimal or absent. Furthermore, to validate the differential binding of CD300a-Ig and CD300c-Ig to PS and PE, we performed Surface Plasmon Resonance (SPR) experiments by immobilizing PC, PS and PE liposomes on L1 chip. Consistent with the results above, we observed that CD300a-Ig predominantly binds to PE liposomes, while CD300c-Ig binds to PS and PE liposomes to a similar extent. Interestingly, this is similar to the exhibited binding of CD300a-Ig to PS (Fig. 5C,D). Altogether, these results indicate that the paired receptors CD300a and CD300c have different binding characteristics to PS and PE.

### PS and PE differentially regulate the function of CD56^bright^ NK cells

**Figure 6:**
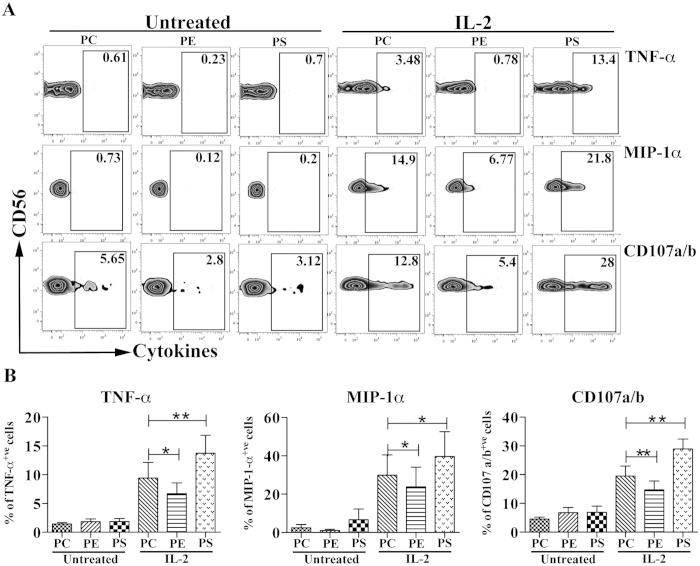
Purified NK cells were both left untreated or pre-treated with IL-2 and cross-linked with pure lipids coated on a plate as mentioned in Materials and Methods. (**A**) The zebra plots represent the secretion of various cytokines and degranulation markers (CD107a/b) from CD56^bright^ NK cells, gated as in Fig. 1 (gated as CD56^bright^ CD16^neg^) stimulated under the indicated conditions. (**B**) Bar graphs represent the percentage of TNF-α, MIP-1α and CD107a/b positive CD56^bright^ NK cells. The data are from independent experiments from 4 donors. The error bars indicate the average ± SEM.

Next, our focus was to understand the functional consequences of NK cells in response to the interaction with the lipids PC, PS and PE. NK cells were purified and were either untreated or pre-treated with recombinant IL-2. As expected, in this set of experiments, we observed a small number of TNF-α^+^ and MIP-1α^+^, as well as degranulating (CD107a/b) IL-2 pre-activated CD56^bright^ NK cells when they were cultured on plates coated with the negative control lipid PC, whereas the IL-2 non-stimulated cells did not show any effector function. Interestingly, IL-2 pre-stimulated NK cells on plates coated with PE showed a significant decrease of cytokine production and degranulation, indicating that PE is an inhibitory ligand (Fig. 6A,B). On the other hand, PS stimulation of IL-2 pre-activated NK cells significantly enhanced the number of cytokine producing and degranulating NK cells, indicating that PS acts as an activating ligand (Fig. 6A,B). These results suggest that the interaction of PE with CD300a is predominant over the interaction with CD300c, which produces an inhibitory signal as a result. In addition, crosslinking of CD300c by PS on IL-2 pre-activated NK cells overrides the crosslinking of CD300a, resulting in a positive signal.

## Discussion

NK cell responses are balanced by a multitude of activating and inhibitory receptors^5^. The receptors expression and function is regulated by various cytokine stimuli^5^. In this study, we investigated the expression pattern of CD300c, an activating receptor that belongs to the CD300 family, which recognizes lipids as their ligands. Signaling through IL-2 and IL-15 induced the expression of CD300c on CD56^bright^ NK cells but not on the CD56^dim^ population in a mechanism that involves STAT5. Interestingly, IL-4, a potent regulator of IL-2 mediated activation of NK cells^41^, inhibits IL-2/IL-15 dependent expression of CD300c on CD56^bright^ NK cells, which is consistent with the ability of IL-4 to inhibit IL-2 triggered STAT5 activation on human T cells^38^.

In humans, the CD56^bright^ NK cell subset has been shown to play an important role in adaptive immunity^15^. CD56^bright^ NK cells, being the most abundant NK cell population in the secondary lymphoid organs, have the capacity to produce abundant cytokines and are thus considered to possess a regulatory role during adaptive immune responses (9, 12, 20). Throughout NK cell development in the secondary lymph nodes, a CD34^+^ precursor population has been identified and shown to differentiate into CD56^bright^ NK cells upon stimulation with either IL-2 or IL-15. Further, these cells reside in the T cell enriched regions of the lymph nodes^16^. Since antigen-activated T cells secrete IL-2, we postulate that the neighboring CD56^bright^ NK cells up-regulate CD300c expression on their surface as a consequence of T cell activation. The data from the transwell assays validate the role of activated CD4+ T cells derived IL-2 in the induction of CD300c on CD56^bright^ NK cells.

It has previously been demonstrated that crosslinking of CD300c activates human monocytes to induce pro-inflammatory responses^35^,^42^. In this report, we show that an antibody against CD300c can induce cytokine secretion and degranulation only in IL-2 pre-activated CD56^bright^ NK cells. The induction of TNF-α, MIP1-α and degranulation (CD107a/b) by IL-2 pre-activated CD56^bright^ NK cells were strongly enhanced after crosslinking of CD300c. On the other hand, IFN-γ induction was minimally increased after CD300c cross-linking on IL-2 pre-activated CD56^bright^ NK cells. However, CD300c mediated signals synergized with IL-12 and IL-18 to produce high amounts of IFN-γ on IL-2 pre-activated cells. Recently, it has been shown that the crosstalk between NK cells and other myeloid cells, especially dendritic cells and macrophages, mutually benefit by reciprocal activation. Cytokines derived from myeloid cells, such as IL-12, IL-15 and IL-18, activate NK cells. Post activation, NK cells secrete pro-inflammatory cytokines like TNF-α that initiate DC maturation and IFN-γ that activates inflammatory macrophages^7^,^11^. In this particular study, we envisage that the unique expression and function of CD300c on CD56^bright^ NK cells might play an important co-stimulatory role in the secondary lymphoid organs in the presence of endogenous T-cell derived IL-2 and cytokines (IL-12, IL-15 and IL-18) derived from antigen presenting cells (APCs).

Among the myriad of activating and inhibitory receptors on NK cells, the paired ones possess highly homologous sequences in the extracellular domain and have opposing functions in immune regulation^43^. Some of the best-described examples are the killer cell immunoglobulin-like receptors (KIR) family and C-type lectin receptor family that recognize MHC Class I molecules^44^. Our data also demonstrated recognition of same ligands by the paired receptors CD300a and CD300c, although with different affinities. PE and PS are the lipids that are exposed on the outer leaflet when the cell membrane is compromised^40^. In line with our previous reports, our current studies demonstrated binding of CD300a to dead cells and the corresponding amino-phospholipids PE and PS. A similar phenomenon was observed for the activating receptor CD300c. The intriguing feature is that the strength of binding between the two receptors to dead cells and PE is very different. CD300a exhibited a stronger binding to dead cells and to PE than CD300c. On the other hand, both CD300a and CD300c exhibited similar binding to PS. Our observaions are also supported and published by Takahashi *et al*.^42^. Therefore, it is acceptable to conclude that the ligand interactions to the activating receptor CD300c are weaker than to its inhibitory counterpart CD300a. The above phenomena are consistent with other paired receptors belonging to different families. For example, KIR2DL1 (KIR family) and CD94/NKG2A (C-type lectin family) have higher binding affinity to HLA-C^Lys80^ and HLA-E, respectively, than their activating counter parts KIR2DS1 and CD94/NKG2C^43^.

Our studies described the ability of PS to induce cytokine production and degranulation by CD56^bright^ NK cells. Considering that PS is not only exposed on apoptotic cells, but also on activated T cells, B cells and monocytes^45^,^46^,^47^, it might engage CD300c on CD56^bright^ NK cells. Predominantly CD56^bright^ NK cell subset has the ability to lyse autologous activated T cells compared to CD56^dim^ NK cell subset^48^,^49^. Moreover, it has been shown that tonsilar CD56^bright^ NK cells can influence T cell polarization during primary immune responses by secretion of IFN-γ^50^. Therefore, it is highly persuasive that CD300c on CD56^bright^ NK cells may facilitate the cross talk between these NK cells and other activated leukocytes. In the past decade, there has also been emerging evidence that CD56^bright^ NK cells restrain T cell responses during various autoimmune diseases^51^. The prognoses of autoimmune diseases is influenced by an activated immune system, such as activated T cells in multiple sclerosis (MS)^52^ and polyclonal activated B cells in systemic lupus erythematous (SLE)^53^. For example, treatment of MS patients with Dacluzimab, an anti-CD25 humanized monoclonal antibody, selectively increases the subset of CD56^bright^ NK cells and is responsible for the cytotoxicity of activated T cells^54^. Others have shown that killing of activated T cells by IL-2 activated NK cells involves the NKG2D, LFA-1 and TRAIL receptors^48^,^49^. Similarly, it may be very possible that CD300c has also an important role, along with other receptors, in the killing of activated T cells.

Contrary to PS activating function, we observed that PE induced a negative response in degranulation and cytokine response of IL-2 pre-activated CD56^bright^ NK cells. Our previous studies^32^ and current data demonstrate that CD300a strongly binds to PE and the binding to PS is weaker, while CD300c binds similarly PE and PS. On the other hand, CD300a binds PE stronger than CD300c, while both receptors similarly bind PS (Fig. 5). While it was expected that interaction with PE would deliver a negative signal, we did not know what to expect when IL-2 (or IL-15) pre-activated CD56^bright^ NK cells interacted with PS. Interestingly, cells de-granulated and produced cytokines in response to PS, indicating that in this scenario the positive signal (CD300c-mediated) overrides the negative signal (CD300a-mediated). It is important to note that the NK cells used in our assays are always pre-activated with IL-2 (or IL-15) to induce CD300c expression on the CD56^bright^ subset. We therefore do not know whether in the absence of pre-activation that interaction with PS would deliver an inhibitory signal. Unfortunately, the experiments cannot be performed with freshly isolated CD56^bright^ NK cells since they do not express (or express very low levels) of CD300c on their cell surface. However, to avoid the co-stimulatory effect of IL-2, cells were rested for 24 hours after cytokine treatment, which may suggest that CD56^bright^ NK cells expressing CD300c do not need to be in a highly activated state to produce cytokines and degranulate after encountering PS.

The finding that two phospholipids, PS and PE, are metabolically related^40^, but differentially regulate the functions of CD56^bright^ NK cells through their interaction with CD300a and CD300c clearly indicate that further studies are necessary to perform in-depth characterization of the immune-regulatory roles of these paired receptors on NK cells. CD300a and CD300c on CD56^bright^ NK cells, with their opposing functions, will modulate the threshold for cell activation after interacting with PE and PS expressing dead and activated cells. Consequently, this might play an important role in maintaining the homeostasis of secondary lymphoid organs during the cross-talk between activated T cells and CD56^bright^ NK cells.

## Methods

### Primary Cells

Leukapheresis blood packs from healthy donors were obtained under an institutional review board-approved protocol at the National Institutes of Health. All donors provided written informed consent. All the cell isolation methods were carried out in accordance with the approved laboratory guidelines. Peripheral blood mononuclear cells (PBMCs) were obtained by ficoll density centrifugation. NK cells were purified from PBMCs using a negative selection method. Naïve CD4^+^ T cells were isolated from PBMCs using a double purification step involving the removal of CD25^+^ regulatory cells and then using a negative selection method for the purification of naïve CD4^+^ T cells. All the kits were purchased from Stem Cell Technologies and isolated using RoboSep. Cells were cultured in Iscove´s Modified Dulbecco´s Medium (IMDM) supplemented with 10% fetal-bovine serum, L-glutamine, sodium pyruvate and non-essential amino acids.

### Antibodies and Reagents

Fluorochrome-labeled antibodies used for flow cytometric analyses were obtained from the following vendors: anti-CD3 (UCHT1), anti-CD16 (eBioCB16), anti-CD56 (CMSSB), anti-CD107a (eBioH4A3), anti-CD107b (eBioH4B4), anti-CD300c (TX45), anti-TNFα (MAb11), anti-MIP1α (PFFM3) and anti-CD69 (FN50) were from eBiosciences and anti-IFN-γ (B27) was from BD Biosciences. Purified antibodies anti-human IL-2 (AB12-3G4), mouse IgG2a κ isotype (eBM2a), mouse IgG1 κ (MOPC-21) and anti-CD300c (TX45) were obtained from eBiosciences. For the real time PCR experiments, primers for CD300A, CD300C and 18sRNA genes were purchased from SA Biosciences. STAT5 inhibitor N′-((4-Oxo-4H-chromen-3-yl)methylene)nicotinohydrazide was purchased from Calbiochem/EMD Millipore. Recombinant IL-2 was obtained from National Cancer Institute, Frederick, MD. Cytokines IL-12, IL-15 and IL-4 are from R&D Systems, IL-18 is from Medical and Biological Laboratories (MBL) and IL-21 is from Peprotech. Phospholipids 1-palmitoyl-2-oleoyl (PO) phosphatidylserine (POPS) (PS), phosphatidylethanolamine (POPE) (PE) and phosphatidylcholine (POPC) (PC) were purchased from Avanti Polar Lipids.

### NK cell Stimulation

Purified NK cells were stimulated with cytokines IL-2 (100–200 U/ml), IL-15 (10 ng/ml), IL-4 (50 ng/ml), IL-12 (10 ng/ml), IL-18 (100 ng/ml) and IL-21 (50 ng/ml) for 40 hours. In the inhibition assays, vehicle DMSO (Sigma) and STAT5 inhibitor were used at 250 nM concentrations during NK cell stimulation. Post incubation, all the respective samples were stained for multiple surface markers and acquired in either LSR-II or Fortessa X-20 (BD Biosciences). Lymphocytes were electronically gated based on forward and side scatter parameters, and NK cells were further gated based on the expression of CD3, CD56 and CD16 (CD3^−^CD56^+^CD16^+/−^). Flow cytometric data were analyzed using FlowJo software (Tree Star).

### Transwell Assays

Purified NK cells were cultured in the inner compartment and the naïve CD4+ T cells were in the outer compartment in a Transwell 24-well plate. The CD4+ T cells were activated with human T-activator CD3/CD28 Dyna beads (Life Technologies). As a control NK cells were also incubated with resting CD4+ T cells in the trans-well system. When indicated, the isotype control IgG2a κ and anti-human IL-2 (eBiosciences) were added at a concentration of 10 μg/ml to the activated T cells compartment. After 2 days of incubation, the NK cells in the inner compartment were observed in a bright field microscope for the cluster formation and later the cells were harvested and analyzed for CD300c surface expression. Recombinant IL-2 (100–200 U/ml) was used as a positive control.

### Functional Experiments

Freshly isolated NK cells were stimulated with recombinant IL-2 for 40 hours, washed thoroughly and starved from IL-2 for 24 hours. For antibody cross-linking experiments, both untreated and IL-2 pre-stimulated NK cells were cross-linked with two different monoclonal antibodies; isotype control (MOPC-21) and anti-CD300c (TX45) plated on a 24-well plate at 10 μg/ml concentrations. For lipid stimulation experiments, the lipids PC, PS and PE were diluted in 100% methanol and air-dried. Later the IL-2 starved NK cells were incubated with the lipid-coated plates. After overnight incubation, the NK cells were stained with appropriate surface receptors and intracellular cytokines. The anti-CD107a/b monoclonal antibodies and monensin (Golgi-stop, a protein transport inhibitor) were added during the culture for the last six hours before harvesting. Subsequently, the cells were acquired in a flow cytometer (BD LSR-II) and analyzed by using FlowJo software (Tree Star).

### Binding Assays

The Ig fusion proteins were constructed, purified and fluorescently labeled as previously described^55^. The cell binding assay protocol was followed as mentioned in our previous report^32^. Briefly, UV-treated Jurkat cells were incubated with the Ig fusion proteins in the presence of binding buffer (10 mM HEPES pH 7.4, 140 mM NaCl, 2.5 mM CaCl_2_ and 2 mM MgCl_2_) for 45 minutes on ice and washed twice with wash buffer (PBS containing 1% of FCS) and re-suspended in PBS for flow cytometric analysis. Cells were also stained with Annexin V-APC (eBiosciences) and 7AAD (Beckmann Coulter) to differentiate between dead and live cells. ELISA binding studies were performed as previously described^32^. Briefly, purified lipids PC, PS and PE were diluted in 100% methanol and air-dried. After subsequent washes and blocking, the wells were incubated with different human Ig fusion proteins, washed and incubated with anti-human Ig (Fcγ specific) antibody coupled to horseradish peroxidase HRP (Jackson Immuno Research) for one hour at RT. The peroxidase activity was analyzed by using TMB substrate (ImmunoPure TMB Substrate Kit-Pierce) and the absorbance was measured at 450 nm in a spectrophotometer. The Surface Plasmon Resonance (SPR) analysis protocol was followed as described earlier and performed in Biacore T200^32^. Briefly, the liposomes composed of various lipids PC, PS and PE were captured on the L1 sensor chip and the human-Ig fusion proteins were injected and carried in flow to study their binding to liposomes. BIACORE T200 Control Software and BIAevaluation Software were used to analyze the SPR experiments.

### Statistical analysis

Data were analyzed using GraphPad Prism software. The data were plotted as bar graphs representing the average ± standard error of the mean (SEM). Pair wise comparisons were examined by a paired Student’s *t*-test. NS: not significant; *P < 0.05, **P < 0.01 ***P < 0.001.

## Additional Information

**How to cite this article**: Dimitrova, M. *et al*. CD300c is uniquely expressed on CD56^bright^ Natural Killer Cells and differs from CD300a upon ligand recognition. *Sci. Rep.* **6**, 23942; doi: 10.1038/srep23942 (2016).

## Supplementary Material

Supplementary Information
