# Supplementary material for: CD300c is uniquely expressed on CD56bright Natural Killer Cells and differs from CD300a upon ligand recognition
Source: Sci Rep. 2016 Apr 4;6:23942. doi: 10.1038/srep23942 (PMC4819222; doi:10.1038/srep23942)
Supplement: Supplementary Information [file srep23942-s1.pdf]

CD300c is uniquely expressed on CD56^{bright} Natural Killer Cells and differs from CD300a upon ligand recognition.

Milena Dimitrova[a], Olatz Zenarruzabeitia[b,c], Francisco Borrego[b,c,d] and Venkateswara R. Simhadri[a,1].

[a]Division of Biotechnology Review and Research-I, Office of Biotechnology Products Review and Research, CDER, Food and Drug Administration, USA. [b]Immunopathology Group, BioCruces Health Research Institute, Barakaldo, Spain. [c]Immunotherapy Group, Basque Center for Transfusion and Human Tissues, Galdakao, Spain. [d]Ikerbasque, Basque Foundation for Science, Bilbao, Spain.

[1]Corresponding author: Dr. Venkateswara R. Simhadri, U.S. Food and Drug Administration, U.S.A. E-mail: venkateswara.simhadri@fda.hhs.gov

Supplementary Information

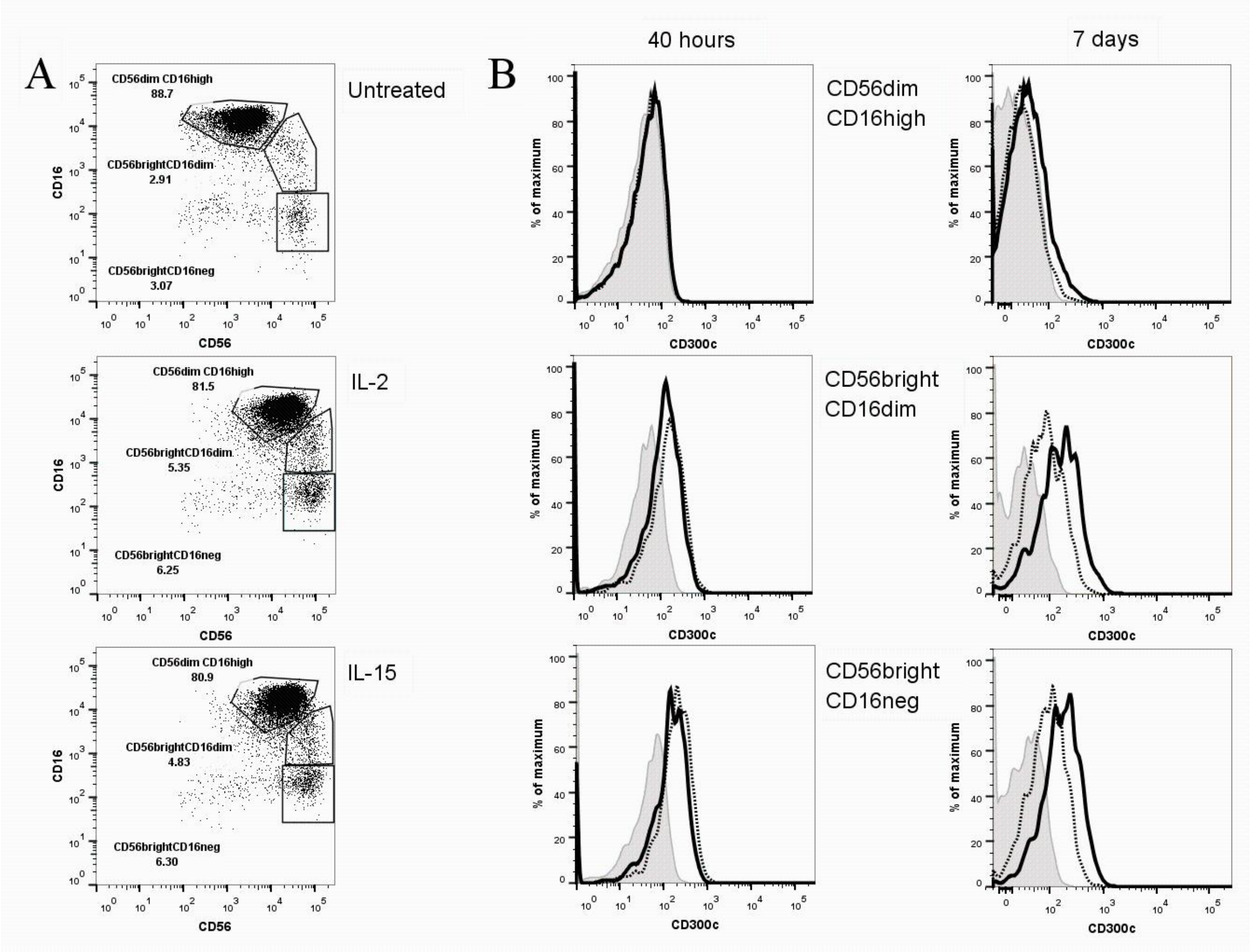

Figure legend: Human NK cells were purified from healthy donor and stimulated with the cytokines IL-2 and IL-15. Flow cytometric analyses were performed after 40 hours and 7 days of stimulation.

(A) Gating strategy of all the three conditions (untreated, IL-2 and IL-15) showing the three different subsets of NK cells (CD56^{dim}CD16^{high}, CD56^{bright}CD16^{dim} and CD56^{bright}CD16^{neg}).

(B) Expression of CD300c on various the three NK cell subsets at 40 hours (left column) and 7 days (right column). Shaded histograms (untreated), thick black line (IL-2 treated) and dotted black line (IL-15 treated).
